# Do consumers ‘Get the facts’? A survey of alcohol warning label recognition in Australia

**DOI:** 10.1186/s12889-015-2160-0

**Published:** 2015-08-22

**Authors:** Kerri Coomber, Florentine Martino, I. Robert Barbour, Richelle Mayshak, Peter G. Miller

**Affiliations:** School of Psychology, Faculty of Health, Deakin University, Locked Bag 20001, Geelong, 3220 Australia

## Abstract

**Background:**

There is limited research on awareness of alcohol warning labels and their effects. The current study examined the awareness of the Australian voluntary warning labels, the ‘Get the facts’ logo (a component of current warning labels) that directs consumers to an industry-designed informational website, and whether alcohol consumers visited this website.

**Methods:**

Participants aged 18–45 (unweighted *n* = 561; mean age = 33.6 years) completed an online survey assessing alcohol consumption patterns, awareness of the ‘Get the facts’ logo and warning labels, and use of the website.

**Results:**

No participants recalled the ‘Get the facts’ logo, and the recall rate of warning labels was 16 % at best. A quarter of participants recognised the ‘Get the facts’ logo, and awareness of the warning labels ranged from 13.1–37.9 %. Overall, only 7.3 % of respondents had visited the website. Multivariable logistic regression models indicated that younger drinkers, increased frequency of binge drinking, consuming alcohol directly from the bottle or can, and support for warning labels were significantly, positively associated with awareness of the logo and warning labels. While an increased frequency of binge drinking, consuming alcohol directly from the container, support for warning labels, and recognition of the ‘Get the facts’ logo increased the odds of visiting the website.

**Conclusions:**

Within this sample, recall of the current, voluntary warning labels on Australian alcohol products was non-existent, overall awareness was low, and few people reported visiting the DrinkWise website. It appears that current warning labels fail to effectively transmit health messages to the general public.

## Background

Globally, alcohol is the third highest cause of disease and disability, and 4 % of deaths worldwide can be attributed to alcohol [1]. Eighty four percent of the Australian adult population are regular consumers of alcohol [2]. Approximately one in five Australians aged 14 years or older drink at levels that put them at risk of harm over their lifetime [2] and more than a quarter of Australian adults consume alcohol at least once a month at levels that put them at risk of acute accident or injury [3]. Despite the patterns of alcohol consumption in Australia, 78 % of adults believe there is a problem of excess drinking or alcohol abuse within society [4]. However, current policies fail to adequately address the full extent of the problem. In line with successful tobacco control measures, recommendations have been made to apply mandatory warning labels on alcohol products, at point of sale, and on advertising to provide much needed health information for drinkers [5–8]. Warning labels, in the context of a comprehensive set of interventions, have the potential to inform consumers of the likely harms of risky drinking, and how consumers might reduce this risk [7, 9, 10].

The comprehensive literature base for tobacco warning labels indicates that such an intervention can be highly successful at changing the attitudes and behaviours of smokers. Tobacco warning labels increase health knowledge and perceptions of risk, aid cessation, and help to prevent smoking initiation [11]. Tobacco label literature also provides transferable knowledge of the key aspects that make warning labels effective, including: position on the label, size of warnings, message type, and warning refreshment [11]. Using tobacco labelling as a ‘best practice’ base [7], there is potential to develop alcohol warning labels that achieve similar outcomes.

**Fig. 1:**
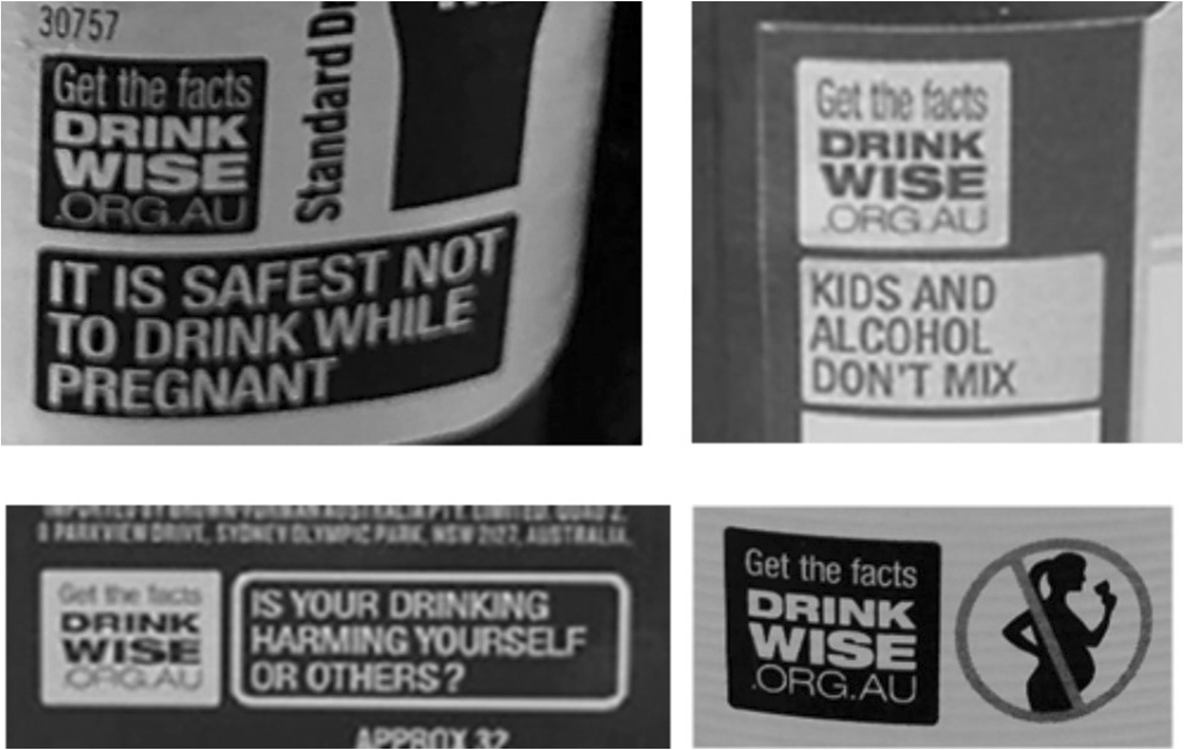
Examples of current voluntary DrinkWise warning labels as used on alcohol products

In July 2011, DrinkWise – an alcohol industry ‘social aspects/public relations’ organisation [12, 13] – implemented new voluntary consumer messages on alcohol products. The introduction of these messages were in response to the recommendation by an independent government review that all alcohol product labels contain a health warning [14]. These voluntary labels depict the core message of ‘Get the facts’, which encourages drinkers to visit the DrinkWise website to research the harms of drinking. According to information on the DrinkWise website, their website contains evidence-based information on alcohol that is designed to help communities take a healthier and safer approach to alcohol consumption. However, research indicates that DrinkWise and other international industry funded organisations, do not actually promote evidence-based interventions and alcohol-harm reduction strategies known to reduce alcohol-related harms [13, 15]. In conjunction with the ‘Get the facts’ logo, alcohol products may also include one of four messages or images: ‘It is safest not to drink while pregnant’; an image of a silhouette of a pregnant woman with a strike through; ‘Is your drinking harming yourself or others?’; or, ‘Kids and alcohol don’t mix’ (see Fig. 1 for examples of these warning labels alcohol products).

The most recent audit of Australian alcohol warning labels showed that these labels are only depicted on approximately one in three alcohol products [16]. Further, current Australian warning labels have been criticised as being simply too small (less than 5 % of the label), being located on the back of the label, utilising vague wording and images, and lacking visual impact to generate an emotional response [9, 17–20]. There is a lack of research examining the effectiveness of alcohol warning labels within the context of alcohol policy more widely. However, for warning labels to be most effective the messages conveyed within the label should be linked with other prevention initiatives, such as alcohol control advertising [7]. Such an approach would increase the exposure of the message and act to reinforce the messages on labels.

Studies on the effectiveness of alcohol warning labels have mostly focused on the mandatory text-based warnings used in the United States (US), with most research being quite dated [21, 5, 22, 7]. After the introduction of the US alcohol text warnings in 1989, there was a steady increase in free recall of these warnings from 3.8 % in 1989–28.5 % in 1993–1994 [23]. More recent research indicates that approximately 20 %–30 % of US adults recall the drink driving text warning [24]. However, these text-based warnings have minimal impact on behaviour change [7, 21]. Awareness of the mandatory US warning labels is highest amongst the youngest group of drinkers (18–29 years) and heavy drinkers due to the likelihood of greater exposure to the containers, and therefore the labels on them [25, 23]. However, these frequent users of alcohol find warning labels relatively less believable, suggesting that the warning label may be partially ignored or discounted by those that need the warning labels the most [26]. Additionally, those with a higher levels of education are more likely to freely recall warning labels [23], but there is no difference by education for prompted recognition of the label [25].

To date, there has been very limited research examining awareness of warning labels on Australian alcohol products. One recent report found that one-third of women were aware of the pregnancy silhouette warning label, and 20 % were aware of the text-based pregnancy label [27]. However, there has been no research investigating whether consumers are aware of the ‘Get the facts’ logo, the other voluntary warning label messages, or if consumers visit the DrinkWise website. Therefore, the aim of the present study is evaluate awareness of the ‘Get the facts’ logo and alcohol warning labels, and to also evaluate consumer use of the DrinkWise website. Demographic predictors of awareness of the logo, warning labels, and use of the DrinkWise website will also be explored.

## Methods

### Participants

Participants were recruited using an online research panel (MyOpinions). In order to capture a wide range of demographics, panel members are recruited by MyOpinions through both online (e.g., banner ads, search engines) and offline (e.g., radio and print advertising) channels. Regular profiling of the online panel ensures that the demographic spread of panel members is representative of the Australian population. Quotas for data collection were set for gender (50/50 %). A total of 1676 respondents commenced the survey. Of these, 885 participants were screened out of the survey due to being outside of the target age range of 18–45 years (*n* = 869) or indicating that they never consumed alcohol (*n* = 16). An additional 230 participants dropped out of the survey at varying points after commencement. Due to the non-random nature of this missing data (i.e., participants chose to discontinue the survey) we opted not to replace this missing data and these responses were excluded. The final sample comprised 561 participants (weighted *n* = 555). Table 1 provides details of the demographics and predictor variables.

**Table. 1:** Characteristics of participants

	Unweighted data		Weighted data^a^	
	N	%	N	%
Gender				
Male	295	52.6	277	49.8
Female	266	47.4	279	50.2
Age				
18–24	98	17.5	132	23.7
25–34	190	33.9	200	35.9
35–45	273	48.7	224	40.4
Education				
Less than tertiary	317	56.5	314	56.6
Tertiary or above	244	43.5	241	43.4
Frequency of binge drinking				
Never	185	33.0	180	32.5
Less than monthly	240	42.8	246	44.3
Monthly	85	15.2	83	15.0
Weekly	43	7.7	39	7.0
Daily or almost daily	8	1.4	7	1.2
Main alcoholic drink				
Beer	166	29.6	153	27.6
Wine	155	27.6	150	27.1
Spirits	115	20.5	119	21.5
Pre-mix	86	15.3	96	17.2
Cider	20	3.6	19	3.5
Other	19	3.4	17	3.1
Drink directly from can or bottle				
Never/not often	170	30.3	168	30.2
At least sometimes	391	69.7	388	69.8
Support for health warning labels				
Neutral or opposed	114	20.3	110	19.8
Support/strongly support	447	70.7	445	80.2

^a^Data weighted by age, sex, and state of residence

### Measures

#### Recall and recognition of the ‘Get the facts’ logo and alcohol warning labels

Participants were first asked how often they see warning labels on alcohol containers, followed by an open-ended item asking ‘Which label(s) do you recall seeing?’ Participants then typed in a brief description of the label to generate a measure of spontaneous warning label recall. Therefore, recall is defined as the spontaneous recall of a warning label in the absence of prompts or cues. The items assessing recall of a warning label were mandatory; that is, participants were unable to proceed in the survey until these items had been answered in order to prevent viewing warning label images that are presented later in the survey. Participant descriptions of the labels were categorised according to each of the warning labels and the ‘Get the facts’ logo. All descriptions that mentioned pregnancy were coded in the one category; that is, we did not code the three pregnancy labels separately.

To assess logo and warning label recognition, the ‘Get the facts’ logo and each label were then shown to participants. Participants were asked if they had seen this logo/label on any alcohol products. Thus, recognition is defined as participants identifying the logo/labels that they have previously seen on alcohol products, after exposure to images of the logo and labels. The spontaneous recall and prompted recognition responses were then combined to generate a measure of overall awareness. Awareness of a label was defined as the proportion of participants who freely recalled the warning label, plus the proportion who of participants who did not freely recall the label, but recognised it after being presented with an image of the label.

#### Use of the DrinkWise website

One item asked if participants had ever visited the DrinkWise website depicted in the ‘Get the facts’ logo and on each alcohol warning label (response options ‘yes’ or ‘no’).

#### Alcohol use

The binge drinking item from the Alcohol Use Disorders Identification Test (AUDIT-C) was used to measure frequency of short-term risky drinking occasions [28]. Items from the AUDIT-C have been validated for use in the general adult population [29, 30]. This item asked ‘How often have you had 6 or more units if female, or 8 or more units if male, on a single occasion in the last year?’ (0 ‘never’ to 4 ‘daily or almost daily’).

Participants were also asked to select which type of alcoholic drink was their main drink of choice. Main alcoholic drink was categorised into beer, wine, spirits, pre-mix, cider, and other (consisting of unspecified alcoholic beverages and home-brew beer). Additionally, participants were asked how often they consume alcohol directly from the can or bottle; responses were dichotomised into ‘sometimes, often, or very often’ compared to ‘not often, or never’.

#### Demographics

Data were collected on sex, age (categorised as 18–24 years, 25–34 years and 35–45 years for analysis), and highest educational attainment (coded as ‘less than tertiary’ or ‘tertiary or higher’). Lastly, participants were asked how strongly they support the use of health warning labels on all alcohol beverages; responses were dichotomised into ‘support or strongly support’ compared to ‘neither support or oppose, oppose, or strongly oppose’.

### Procedure

The study was approved by the Human Research Ethics committee of Deakin University. Participants who were part of an opt-in panel were contacted about the study via email, with data collection via an online survey using SurveyMonkey. The email contained the plain language statement and a link to the survey. The online survey panel monitors survey completion by panel identification number, therefore, panel members are precluded from completing the survey more than once. Participants indicated consent to complete the survey by clicking on the survey link. Survey completion time was between 10–15 min.

### Data analysis

All analyses were undertaken using Stata 12.1 [31] and post-stratification population weights were applied (using the *svy* command with ‘p’ weights) that accounted for age, gender, and state of residence using 2011 Australian Census Data [32]. Multivariable logistic regression analyses were used to examine predictors of recognition of the Get the Facts logo, awareness of each of the warning labels, and visiting the DrinkWise website. Prior to analyses, multicollinearity between the independent variables was tested using pairwise correlations and the *collin* command. Correlations between predictor variables were low (maximum *r* = 0.27), and the variance inflation factors were also low (mean = 1.09; range = 1.04–1.15), indicating no issues with multicollinearity [33].

## Results

Table 2 shows that no participants freely recalled the ‘Get the facts’ logo, while a quarter of participants recognised this logo. Participants had the highest rate of overall awareness of the pregnancy warning labels, just under one-fifth of participants stated they had awareness of ‘Is drinking harming yourself or others?’ and approximately 13 % of participants were aware of ‘Kids and alcohol don’t mix’.

**Table. 2:** Weighted proportion of respondents who freely recalled or recognised each alcohol warning label, the ‘Get the facts’ logo, and overall awareness

	Recall	Recognition	Overall awareness^a^
‘Get the facts’ logo	0 %	25.3 %	25.3 %
It is safest not to drink while pregnant^b^	16.1 %	34.3 %	37.9 %
Is your drinking harming yourself or others?	1.5 %	18.2 %	19.5 %
Kids and alcohol don’t mix	0.4 %	12.9 %	13.1 %

^a^Overall awareness takes into account both recall and recognition

^b^Recall, recognition, and awareness of any of the three pregnancy warning labels

*Note.* Data weighted by age, sex, and state of residence

### Recognition of the ‘Get the facts’ logo

Table 3 provides results for the multivariable logistic regression examining predictors of recognition of the ‘Get the facts’ logo. The following factors were associated with a significantly increased odds of recognising the logo: more frequently engaging in binge drinking; consuming alcohol directly from a can or bottle at least some of the time; and, supporting the use of health warning labels. Older participants were significantly less likely than 18–24 year olds to recognise the logo. No other significant demographic differences were found.

**Table. 3:** Results of logistic regression models predicting recognition of the ‘Get the facts’ logo

	OR (95 % CI)	p
Gender		
Male	1.00	
Female	0.65 (0.40–1.07)	.090
Age	Wald *χ* ^2^ *p* < .001	
18–24	1.00	
25–34	0.85 (0.47–1.56)	.610
35–45	0.35 (0.19–0.65)	.001
Education		
Less than tertiary	1.00	
Tertiary or above	0.94 (0.59–1.51)	.800
Binge drinking	1.61 (1.29–2.00)	<.001
Main alcoholic drink	Wald *χ* ^2^ *p* = .358	
Beer	1.00	
Wine	1.20 (0.65–2.22)	.551
Spirits	0.68 (0.33–1.38)	.281
Pre-mix	0.60 (0.28–1.29)	.191
Cider	0.79 (0.21–2.92)	.726
Other	1.56 (0.56–4.40)	.397
Drink directly from can or bottle		
Never/not often	1.00	
At least sometimes	1.75 (1.02–3.01)	.041
Support for health warning labels		
Neutral or opposed	1.00	
Support/strongly support	1.92 (1.05–3.53)	.034

*Note.* Data weighted by age, sex, and state of residence

### Overall awareness of the warning labels

The predictors of awareness of warning labels were largely consistent with those found for recognition of the logo. Participants who engaged in more frequent binge drinking, those who consumed alcohol directly from a can or bottle, and participants who supported the use of health warning labels were all significantly more likely to be aware of alcohol warning labels (see Table 4). Older participants were significantly less likely than 18–24 year olds be aware of any of the warning labels. For the ‘Kids and alcohol don’t mix’ label, 25–34 year olds were significantly less likely to be aware of this label, while those with a higher level of education were significantly more likely to be aware of this label. No other significant differences for awareness of warning labels were found.

**Table. 4:** Results of logistic regression models predicting awareness of alcohol warning labels

	It is safest not to drink while pregnant^a^		Is your drinking harming yourself or others?		Kids and alcohol don’t mix	
	OR (95 % CI)	p	OR (95 % CI)	p	OR (95 % CI)	p
Gender						
Male	1.00		1.00		1.00	
Female	1.22 (0.78–1.90)	.391	0.77 (0.44–1.34)	.350	0.58 (0.29–1.16)	.125
Age	Wald *χ* ^2^ *p* < .001		Wald *χ* ^2^ *p* = .026		Wald *χ* ^2^ *p* = .004	
18–24	1.00		1.00		1.00	
25–34	0.97 (0.56–1.64)	.873	0.56 (0.29–1.11)	.096	0.40 (0.19–0.84)	.016
35–45	0.31 (0.18–0.54)	<.001	0.41 (0.21–0.78)	.007	0.29 (0.14–0.60)	.001
Education						
Less than tertiary	1.00		1.00		1.00	
Tertiary or above	0.95 (0.62–1.46)	.818	1.57 (0.92–2.68)	.095	1.97 (1.02–3.84)	.045
Binge drinking	1.40 (1.13–1.72)	.002	1.55 (1.20–2.00)	.001	1.54 (1.15–2.05)	.004
Main alcoholic drink	Wald *χ* ^2^ *p* = .204		Wald *χ* ^2^ *p* = .250		Wald *χ* ^2^ *p* = .069	
Beer	1.00		1.00		1.00	
Wine	1.23 (0.70–2.16)	.472	1.72 (0.89–3.31)	.108	2.51 (1.04–6.06)	.041
Spirits	0.88 (0.48–1.61)	.671	1.36 (0.65–2.85)	.418	1.16 (0.42–3.20)	.776
Pre-mix	0.48 (0.24–0.98)	.045	0.63 (0.25–1.59)	.325	0.81 (0.25–2.67)	.740
Cider	0.82 (0.28–2.39)	.718	1.41 (0.34–5.85)	.640	1.38 (0.23–8.20)	.725
Other	0.95 (0.30–2.97)	.929	1.19 (0.31–4.56)	.796	4.22 (1.22–14.68)	.023
Drink directly from can or bottle						
Never/not often	1.00		1.00		1.00	
At least sometimes	1.75 (1.10–2.77)	.018	2.24 (1.21–4.13)	.010	2.90 (0.93–3.88)	.077
Support for health warning labels						
Neutral or opposed	1.00		1.00		1.00	
Support/strongly support	2.48 (1.41–4.37)	.002	2.53 (1.25–5.12)	.010	2.65 (1.06–6.60)	.037

^a^Awareness of any of the three pregnancy warning labels

*Note.* Data weighted by age, sex, and state of residence

### Use of the drinkwise website

Forty (7.3 % weighted) participants reported having visited the DrinkWise website. Of those 40 participants, 32 (80.4 %) visited the website due to seeing the logo, whereas eight (19.6 %) visited it for other reasons. An important predictor of visiting the website was recognition of the ‘Get the Facts’ logo; those who recognised the logo were over seven times as likely to visit the website as those who did not recognise the logo (see Table 5). Females were significantly less likely than males to have visited the DrinkWise website. In addition, wine drinkers and spirits drinkers were significantly more likely than beer drinkers to visit the website. As per the findings for awareness of the warning labels, more frequent binge drinkers, participants who consumed alcohol directly from a can or bottle, and those who supported the used of health-focussed warning labels were all significantly more likely to have been to the DrinkWise website.

**Table. 5:** Results of logistic regression models predicting visiting the DrinkWise website

	OR (95 % CI)	p
Gender		
Male	1.00	
Female	0.23 (0.08–0.63)	.004
Age	Wald *χ* ^2^ p = .304	
18–24	1.00	
25–34	0.80 (0.27–2.38)	.682
35–45	0.46 (0.15–1.42)	.176
Education		
Less than tertiary	1.00	
Tertiary or above	1.27 (0.50–3.26)	.613
Binge drinking	1.56 (1.07–2.78)	.022
Main alcoholic drink	Wald *χ* ^2^ p = .004	
Beer	1.00	
Wine	10.25 (3.35–33.36)	<.001
Spirits	6.23 (1.57–24.73)	.009
Pre-mix	0.77 (0.08–7.87)	.827
Cider	2.45 (0.16–38.05)	.520
Other	2.78 (0.24–32.27)	.413
Drink directly from can or bottle		
Never/not often	1.00	
At least sometimes	3.50 (1.11–11.02)	.032
Support for health warning labels		
Neutral or opposed	1.00	
Support/strongly support	4.27 (1.09–16.80)	.038
Recognition of the ‘Get the facts’ logo		
No	1.00	
Yes	7.25 (2.50–21.01)	<.001

*Note.* Data weighted by age, sex, and state of residence

## Discussion

This is the first study to explore the factors that influence awareness of Australian alcohol warning labels. This study is also the first to determine predictors of consumers visiting the DrinkWise website. Frequency of binge drinking, drinking directly from a can or bottle, and support for warning labels were all found to have significant positive associations with recognition of the logo, awareness of the warning labels, and visiting the DrinkWise website. However, older drinkers and females were less likely to be aware of the warning labels and visit the website, respectively.

The rate of logo and warning label recall in the current study was five to 28 percentage points lower than the recall of the mandatory text warnings in the US [23, 24]. This general low level of warning label recall could be attributable to one-third of Australian alcohol products displaying a warning label, usually taking up less than 5 % of the label and on the back of the product [16]. However, the discrepancy in recall compared to the US studies may also be attributable to the US studies utilising telephone interviews rather than online surveys [23, 24]. Participants in the current study demonstrated the highest rate of awareness for the pregnancy warning labels. This level of awareness is most likely due to the pregnancy silhouette being the most common warning used [16] and pictorial labels being more noticeable [7].

The overall lack of awareness of warning labels may also stem from consumers misunderstanding what constitutes a health warning. Many young drinkers often confuse warning labels with ‘drink responsibly’ messages often displayed on product labels [34]. In our study, 22.8 % (weighted) of participants freely recalled either standard drink information or drink responsibly message when asked to describe a warning label – nearly two times higher than the percentage of participants who freely recalled a pregnancy warning label. While it appears consumers believe ‘drink responsibly’ and standard drink messages to be a warning label, past research indicates that the standard drink information on products often simply enables young adult drinkers to select the strongest drinks for the lowest cost, thus actually encouraging heavy drinking [35].

The current study found that frequency of binge drinking increased the odds of exposure to the logo, labels, and visiting the DrinkWise website. Prior research indicates that high-risk drinking is associated with greater awareness of warning label messages [25, 23]. Additional longitudinal research is required to determine whether the utilisation of the DrinkWise website reduces the frequency of heavy drinking occasions. Younger adults in the current study were also more likely to be aware of the warning labels. Australians aged 18–24 years tend to consume greater quantities of alcoholic beverages than drinkers of any other age group [2]. The higher alcohol consumption amongst this age group would lead to a greater potential of exposure to warning labels, and thus, higher levels of awareness of warning labels than older drinkers [23]. Our findings are also consistent with international research that shows younger people and heavy drinkers have the greatest increase over time for recall of warning label messages, most likely due to a higher exposure to such messages [36, 5].

Some of the higher level of logo and label awareness among young people may also be hypothesised to be due to the increased proportion consuming alcohol directly from a can or bottle. The current study also found that those who consume directly from a can or bottle are more likely to be aware of the logo, labels and visit the website. The majority of 18–25 year olds consume most of their alcohol within their home before going out to licensed venues [37], and it may be that this pre-drinking is more likely to be directly from the bottle or can. Post-hoc analyses indicated that for our study, 18–24 year olds were significantly more likely to drink directly from a container than 35–45 year olds (OR = 2.19; 95 % CI = 1.22–3.93, *p* = .008). Previous research has also shown that students who drink directly from the alcohol container have a more accurate memory for the risks depicted on a warning label than students who poured their beverage into a glass [38]. Recent research indicates approximately 41 % of heavy drinking occasions, and 50 % of low-risk drinking occasions occur within a licensed venue [39]. With alcohol commonly served by the glass at licensed venues, and not in its original container, the likelihood of exposure to an alcohol warning label decreases. Thus, comprehensive warning label policy needs to include the requirement for the use of signs or posters in highly visible locations within licenced venues to reinforce the warning label messages and reach consumers that may not see warnings on alcohol containers. Such a requirement would be in line with the successful current tobacco control policies in Australia [40, 41].

Interestingly, while young adults were more likely to recognise the logo and warning labels they were not more likely to have visited the website. Studies with tobacco smokers have found that, in line with cognitive dissonance theory [42], when confronted with graphic warning labels, some smokers may report more positive cognitions about smoking [43] and rationalise their behaviour by changing their beliefs [44, 45]. It may be theorised that young people are rationalising their drinking behaviour through decreased perceptions of risk [45]; these groups may believe that they do not need to ‘Get the facts’. If alcohol warning labels are to be effective, messages targeted at specific subpopulations need to be developed [46].

While there was a low rate of recognition for the ‘Get the Facts’ logo, those who did recognise the logo were seven times more likely to have visited the DrinkWise website. While the current study asked participants if they learned new information from the DrinkWise website, the number of participants who reported having visited the website was too low to conduct any meaningful analysis. Further research is needed to evaluate the effectiveness of a consumer targeted alcohol control website. There is also a need to ensure that information presented on a consumer information website is evidence-based, useful and provides practical, new advice. Currently, the DrinkWise website is used to create an impression of social responsibility, and does not promote evidence-based interventions and alcohol-harm reduction strategies [13]. For instance, while there is strong evidence that increasing the price of alcohol through increased taxation leads to a decline in consumption, DrinkWise does not support such an intervention [13]. Therefore, the establishment of a website by an independent, non-biased source is required to ensure consumers are provided with accurate and evidence-based information. In order to improve consumer utilisation of such a website, the size of warnings and the frequency with which warning labels appear on products need to increase. Additionally, promotional strategies for an independent alcohol awareness website could be implemented to boost website traffic.

### Limitations

The current study recruited participants using an online research panel, which may limit the external validity of the study. However, in line with recommendations the data have been weighted to be more representative of the population [47]. Additionally, the current study was cross-sectional in design, with longitudinal research required to track changes in cognitions and behaviours of consumers after exposure to warning labels [17]. Further, given the number of analyses conducted, caution must be used when interpreting findings that were not part of a systematic pattern of effects.

## Conclusions

The current study demonstrates low awareness of Australian alcohol warning labels, and lack of consumer use of the industry-funded DrinkWise website. The finding that drinking alcohol directly from the container increases exposure to warnings suggests that the use of warnings in other locations, such as at point of sale and within alcohol advertisements [5–8], will help to reinforce such health warning messages. Given that the majority of the Australian public support the introduction of mandatory health warning labels for alcohol products [46], and the success of tobacco labelling [7], graphic, highly visible alcohol warning labels placed on the front of products have the potential to reduce alcohol consumption and alcohol-related harms [11].

## Abbreviations

US: United States
AUDIT-C: Alcohol Use Disorders Identification Test-C
OR: Odds ratio 95 % CI: 95 % confidence intervals
